# Characteristics of a good training placement in psychiatry: qualitative interview study of core trainees

**DOI:** 10.1192/bjo.2025.10777

**Published:** 2025-08-18

**Authors:** Ioana Varvari, Frances Debell, David Harrison, Gopinath Ranjith

**Affiliations:** South London and Maudsley NHS Foundation Trust, London, UK; Royal College of Physicians, London, UK; University College London, UK

**Keywords:** Medical education, psychiatry, training, perception, placement

## Abstract

**Background:**

Clinical placements are essential in healthcare education, offering practical experience and skill development under experienced supervision. However, little research has explored the characteristics of effective psychiatry placements. Understanding the factors considered vital by psychiatry core trainees for a successful placement is crucial amid concerns about trainee attrition in psychiatry programmes.

**Aims:**

This study aims to identify key elements that contribute to a successful psychiatric placement, as perceived by final-year core trainees.

**Method:**

This qualitative study uses one-hour, semi-structured interviews with 15 core trainees in their final placement within the South London and Maudsley Training Programme. Interviews were guided by appreciative inquiry principles, and two independent researchers employed a classic thematic analysis method while maintaining appropriate reflexivity throughout.

**Results:**

A central theme emerges regarding the importance of a well-designed learning environment, which includes a robust training infrastructure, psychological safety, active learning opportunities, access to role models and structured feedback. The supervisor–supervisee relationship is emphasised, with the ideal supervisor being both knowledgeable and empathetic and offering mentorship and pastoral support. These factors are key to professional growth, well-being and job satisfaction, and they are strongly linked to retention in the field.

**Conclusion:**

Core trainees value placements that address foundational training needs and cultivate psychological safety while facilitating experiential learning. Addressing these aspects in training programmes enhances the educational experience and improves retention. Future research should explore supervisors’ perspectives and examine how to balance ideal and practical supervisory roles.

Clinical placements are indispensable components of healthcare education, providing opportunities to gain practical experience and develop clinical competency under the guidance of experienced professionals. Despite playing an important formative educational role, qualitative research on the characteristics of effective psychiatry clinical placements is limited from a core psychiatry trainee pesrpective. Previous studies, mainly interview studies or surveys of large numbers of trainees, focused on the quality of placements or trainee satisfaction^1,2^ and are therefore not designed to answer the question: what makes a good psychiatry placement? Given this critical gap in the literature, and the concerning trend of trainee attrition from psychiatry schemes,^3^ this study explores key factors considered crucial by trainees for a successful placement, to inform best practice in postgraduate medical education and ultimately contribute to improving the psychiatry trainees’ training experience. An innovative aspect of this study is the use of appreciative inquiry^4^ to elicit trainees’ positive experiences. This provides a unique perspective complementing traditional research methods, by uncovering valuable insights into what works well – aspects often overlooked by a focus on negative aspects, particularly in the context of overall low morale among trainees.^5–8^

## Method

### Study design

This study employs a phenomenological research design to explore the perceptions of psychiatry core trainees of their ‘good psychiatry placement’ experiences. In the UK training pathway, core trainees are trainees in their first 3 years of specialty training (CT1–3), which is distinct from higher-specialty trainees at a later stage (ST4–6). They are broadly equivalent to the early years of a residency elsewhere: doctors are learning foundational skills and rotating through different sub-specialties. The appreciative inquiry method creates a positive and respectful atmosphere for discussing these experiences, and for identifying positive aspects and potential areas for growth.

### Participant selection

Core trainees in their final placement, with a minimum of five different placements in the South London and Maudsley (SLaM) Training Programme during the 2022/2023 academic year, were purposively selected. Thirty eligible trainees were identified, and an active recruitment strategy (email invitations) was employed. Fifteen core trainees responded and were sent an information sheet by the study administrator. Participants expressing interest had a pre-online interview meeting with a researcher to answer any questions and provide written consent.

### Data collection

Single, 1:1, semi-structured online interviews (approximately 45 min long) consisting of 12 open-ended questions were conducted by I.V. and F.D. All participants were offered the choice of face-to-face or online interviews, with 14 preferring online interviews and one preferring a face-to-face interview that was held at the Medical Education Office, Maudsley Hospital. Confidentiality was ensured and, with participant consent, online interviews were video and audio recorded on Microsoft Teams (version 2023 for Windows; Microsoft, Redmond, WA, USA; https://www.microsoft.com/en-us/microsoft-teams/download-app) for online participants (and audio recorded for the face-to-face interviewee). The design of the 12-item interview schedule (see Supplementary material available at https://doi.org/10.1192/bjo.2025.10777) employed appreciative inquiry principles by prompting trainees to tell their stories in order to make sense of and interpret their experiences (e.g. Can you think of your most positive clinical placement experience?), positively framing the questions (e.g. What made that placement positive?) and fostering positive anticipatory imagery (e.g. how these positive experiences can shape your current and future development). By prompting active reflection through follow-up questions, we created opportunities to frame experiences positively and fostered a safe and collaborative environment for participants to air these views. All these align with the constructionist, positive, anticipatory and simultaneity principles^4^ of appreciative inquiry.

### Data analysis

Verbatim transcripts were manually prepared and analysed using a thematic analysis method.^9^ I.V. and F.D. independently conducted analyses. First, they became familiar with the transcripts by repeated reading. Second, they conducted initial coding, systematically identifying and labelling meaningful segments of text, followed by organising them into potential themes. Third, they met to reconcile findings and interpretations into initial themes. Finally, resulting themes were discussed at the peer debriefing meetings, where D.H. and G.R. helped refine and confirm the final thematic structure and a coherent analytic narrative. Although thematic saturation was reached with the tenth online interview, the remaining five online were conducted to strengthen sample size, ensure robust findings and enhance generalisability. Only the first section (seven items) of the interview schedule is presented here, to ensure depth of analysis and authenticity of experiences captured by a general exploration of the research question. The second section will be the focus of a separate paper, highlighting contrasts between trainees’ and governing bodies’ definitions of a good placement.

### Research team and reflexivity

The research team consisted of four members: I.V., F.D., D.H. and G.R. I.V., a postgraduate medical education fellow at SLaM in 2022/2023, completed her core training at a different NHS Trust in 2022. Her external training background offered a degree of distance from SLaM’s specific context and helped challenge assumptions that team members more embedded in the local training environment might otherwise take for granted. F.D., an ST5 trained in SLaM, provided a complementary viewpoint. D.H., an educationalist with a background in medical education research, and not trained as a physician/psychiatrist, contributed an outsider’s perspective to the team. G.R., Director of Postgraduate Psychiatry Training at SLaM, provided extensive experience as a clinical and educational supervisor and medical education leader of nearly 20 years’ experience.

As lead researcher, I.V. acknowledges her background in medical education (currently undertaking a Medical Education Master’s Degree) and how this might shape her perspectives and interpretation of the data. To balance these views, F.D. brings a clinical perspective to data collection and interpretation. Both I.V. and F.D. are closer in training to core trainees and acknowledge that their own recent training experiences might influence their interpretation of the data. To challenge potential bias, individual reflexivity journals were maintained and regular peer debriefing sessions conducted between I.V. and F.D., and also between the entire team, with G.R. and D.H. able to balance with more senior clinical and non-clinical perspectives. These sessions fostered open and honest discussions, allowing us to challenge each other’s assumptions and explore alternative interpretations of data.

Finally, to ascertain the accuracy and resonance of our interpretations, we practised member checking by presenting preliminary findings to a subset of two participants selected based on their diverse experiences, and I.V. conducted an online discussion group to capture their feedback and present it to the wider team.

### Ethical approval

The authors assert that all procedures contributing to this work comply with Health Research Authority Guidelines and the General Data Protection Regulation (GDPR), and the Helsinki Declaration of 1975 as revised in 2013. All procedures involving human participants were approved by the Research & Development Team at SLaM and received HRA ethics approval (IRAS 328698, approval no. 23/HRA/2183).

## Results

The study objective was to delve into the lived experience of psychiatry core trainees and explore positive training experiences. Several key themes emerged that answer the questions: ‘What makes a good clinical placement?’ and ‘Why do trainees value these components and experiences?’.These themes allow us to follow our trainees in their journey, from initial contact with a new placement to their reflections and aspirations at the end of the placement.

For clarity, the report is structured into sections. Each section follows our interview guide, with several core themes. Additionally, specific codes, relevant to providing a meaningful narrative are presented within single quotation mark*s*. Excerpts from the data that exemplify our findings are presented as distinct text blocks.

### Section 1: What makes a good clinical psychiatry placement?

**Fig. 1:**
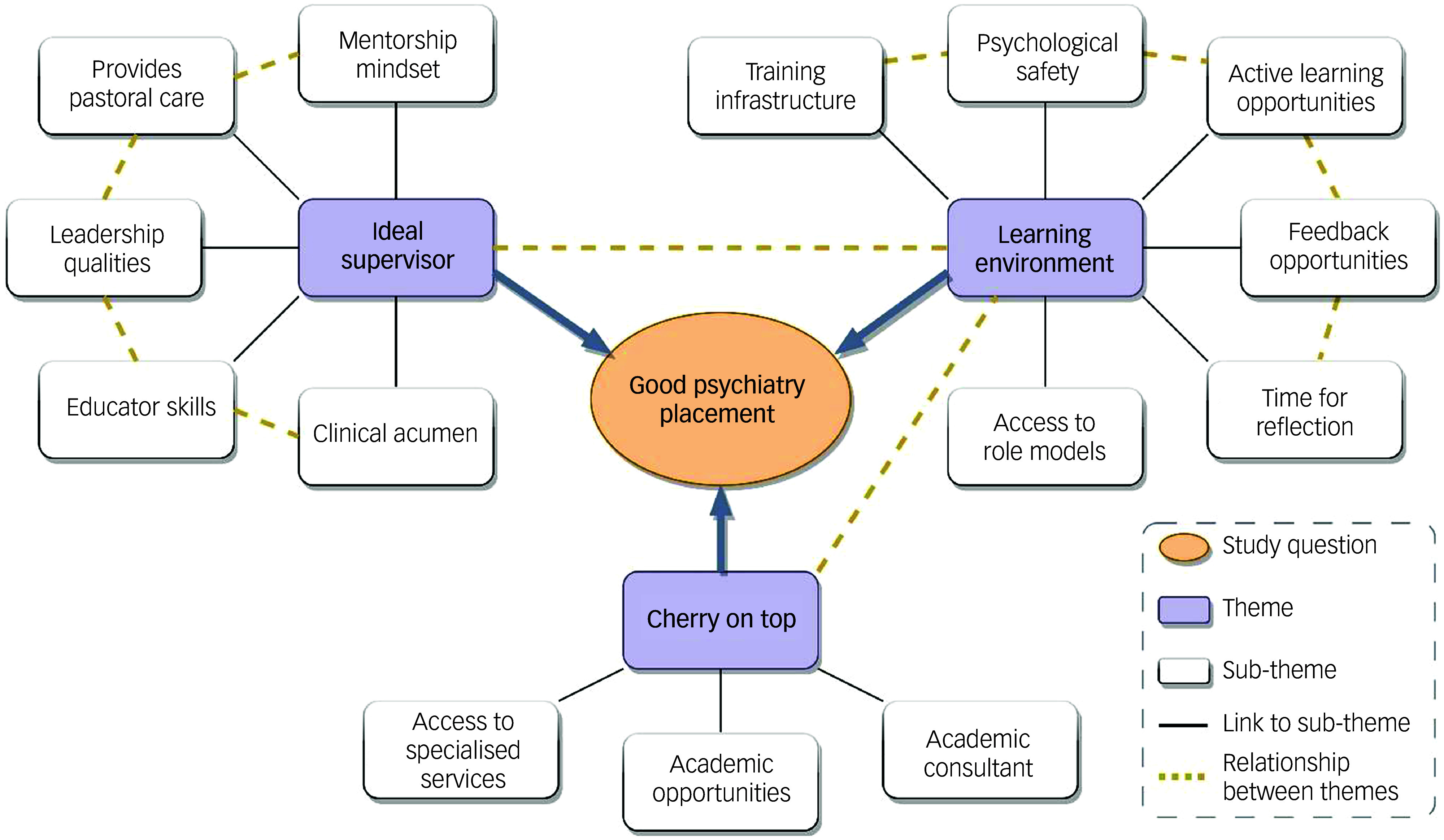
Main themes and sub-themes of ‘What makes a good psychiatry clinical placement?’

Two overarching themes emerged consistently across all 15 interviews: (a) being in a placement that enables learning and (b) having a good supervisor–supervisee relationship. Additionally, a third theme was raised by a few trainees and considered necessary when training at SLaM, which we named (c) ‘the cherry on top’. Main themes and sub-themes are depicted in Fig. 1.

### Theme 1: The placement enables learning

When starting a new placement, trainees ‘take time to observe the team dynamics and interactions’, highlighting the importance of the initial introduction to the team and trainees’ perceptions of the environment: ‘supportive’ versus ‘toxic’. Achieving psychological safety and maintaining it throughout the placement is key in fostering a good training experience. This is linked to a culture of treating core trainees as doctors-in-training where they feel included and recognised, not being asked to shoulder risk alone, and by a clinical supervisor described as ‘genuinely interested’, ‘facilitated the right amount of responsibility’, ‘caring’, ‘provided empathetic feedback’ and ‘provided support early on with challenges’:

‘If we are saying “good placement” … then things like being included in the handovers, in the team gatherings … but, also a communality around jobs and sharing things […] I think teams that have the awareness that it’s nice to be included in the team’s get-togethers make a good placement’.

*‘* … there were jobs where I felt I had the right balance of autonomy and supervision, where I never felt left on my own to do something I did not feel properly trained or uncomfortable with … but, still felt challenged enough …’

With psychological safety achieved, trainees are more likely to benefit from placements, in particular accessing active learning opportunities, which core trainees recounted with excitement and enthusiasm. Placements where trainees ‘had ownership’ over ‘small caseloads’ are highly valued, as are those where they feel ‘intellectually challenged’:

‘My best most positive experience was … where, as a trainee, I was a care coordinator for my patients. I found that positive, because it meant I longitudinally got to follow my patients from the beginning. […] I found that effective and I got to manage my schedule well … […] being able to make those judgements for myself for patients that I know well, as opposed to having my schedule filled with other people’s patients was a different type of challenge, a good one, where I did not feel all over the place’.

Furthermore, having access to ‘a good range of pathologies’ and ‘a variety of experiences’, and being given tasks that ‘were appropriate for their level of training’, are deemed important by the majority of interviewees:

‘… what was good about this placement is the breadth of patients you get to see. EFor example, on the wards, you mainly see patients with psychosis, whereas with the home treatment team, you have more of a mix. And in some ways, it was hard, because they were skills that you had not developed before, but it was therefore really interesting because you’re like “wow, this is just a bit of psychiatry I haven’t done” … and is all supervised and well contained … that was probably one of the best placements’.

Trainees are also more positive about those placements conducive to developing core psychiatry skills rather than focusing on service provision tasks such as ‘taking blood and doing ECGs’:

‘… the team recognised that we are trying to train and that our long goal is to become consultants, and that developing skills is valuable to both us and the team … I think that just the sense of recognising that we aren’t there just to type and take blood and fill in those kinds of spaces … is really important!’

‘[…] So, I think a good placement will be one where these tasks are well supported, where there is a dedicated phlebotomist or nurse to help with that … and then you can spend more time with the patients and practice interviewing them and assess psychopathology … […]’

Two other subthemes, receiving constructive feedback and having the time to reflect, are considered essential components of a successful educational experience. Trainees feel that learning opportunities without these elements make the placement less beneficial and generate ‘uncertainty’ about their skill set:

‘A good clinical placement helps you develop your autonomy by doing things independently and being observed doing those things … because it’s all well and good, you are doing those activities, but if your supervisor never sees you do any of them and discuss them with you, then you are probably learning a bit, but not quite finessing your skills in the same way as the placements where your supervisor is very hand on and watches you do some of those things and then give feedback’.

‘… and then … maybe … the placement not being too hectic, so that there is time within the day to settle and think through what you have seen and what you have done … potentially even do a little bit of reading around it, or even just having your own space to reflect on what’s happened …*’*

Finally, learning is not possible without the right ‘training infrastructure’, covering having the necessary tools through to a placement structured to acknowledge and facilitate learning within the team’s wider processes:

‘… appropriate facilities and equipment is one of the big components … it’s incredibly demoralising to stay late, to be made busy, because you don’t have the necessary infrastructure and equipment to do your job …’

‘I think a really good example is the way the eating disorder placement is run here … most of the job is supporting the in-patient ward, but there is one day a week in out-patients that is completely framed to give trainees a really good clinical learning experience … it is constructed so that you do an assessment in the morning … and then an option to do CBT cases in the afternoon … all followed up by supervisor discussion. […] I thought that was a really well-handled way to balance “we need someone to take bloods and do physical health checks” with “how can we add something to your training and development?”’

### Theme 2: Supervisor–supervisee relationship

Throughout our analysis, a prominent, recurring theme was the intricate and multifaceted dynamics inherent in the supervisor–supervisee relationship. This highlights the pivotal role of this interpersonal connection, and underscores its profound influence on the outcomes, experiences and interactions that make a successful psychiatry clinical placement. Figure 1 visually represents trainees’ perceptions of the ‘ideal supervisor’ and their expectations for this relationship, emphasising the desire for a more connected and mentored experience.

The ideal supervisor is perceived as a blend of exceptional clinical acumen, leadership qualities and educator skills, with a mentorship mindset and someone who provides pastoral care:

‘… I was on an in-patient ward … my consultant was really fantastic! Possibly one of the best I ever had, because he really was not hierarchical and took the time to know the whole MDT and as junior we were taught to value every member of the team … I think you can feel when your supervisor actually wants to be there … […] …also, my supervisor asks me how I am, which I am really not used to, but it feels very nice … he is actually asking about my psychological well-being and recognises that psychiatry can be really, really … heavy. He is just taking a bit of interest in my well-being and how what’s going on in my life might affect work … […] and then his supervision I think is really good … like “let’s get your WPBAs done”, “let’s factor in any exams you have coming up”, but then also, “How are we going to develop you in a professional?”…*’*

‘I have gone through and let every supervisor know that I am dyslexic, and they usually say “it’s okay, thanks for letting us know”, but my last supervisor really thought through what support I might actually need and got me a dictaphone and referred me to occupational therapy … which didn’t know there is an option … I felt very supported by her’.

‘I think having close concurrent mentorship is the most important aspect for sure […] it means that the consultant understands your needs, looks over your CV, what you’ve done so far and what you want to do in the future, what are your gaps and what do you find interesting and would like to explore and sort of takes an interest in you as a person, rather than simply how much clinical work can you do and how good that is? […]*’*

### Theme 3: The cherry on top

Beyond a supportive supervisor and robust learning environment, trainees prize cherry on top opportunities that ‘enrich training experience’, such as ‘access to ‘specialised services’ (i.e. neuropsychiatry clinics, national personality disorder service, women’s health clinics etc.). This was particularly seen as ‘energising’, ‘inspiring’ and ‘broadening clinical acumen’. Furthermore, ‘having an academic consulltant’ and ‘academic opportunities’ were well appreciated by trainees placed in such placements, and desired by the rest. Particularly, consultants with an ‘academic mindset’ were observed to be ‘more likely to help development’ and ‘identify extracurricular opportunities’. Activities such as the ‘elite journal club presentations’ (where experts sit on the discussions panel) and ‘the ground round’ at SLAM were equally appreciated by all trainees. Importantly for the ambitious trainees, academic consultant or placements are more likely to end in ‘published work at a young stage’, which trainees find ‘rewarding’.

### Section 2: ‘Why’ these components are perceived as important

**Fig. 2:**
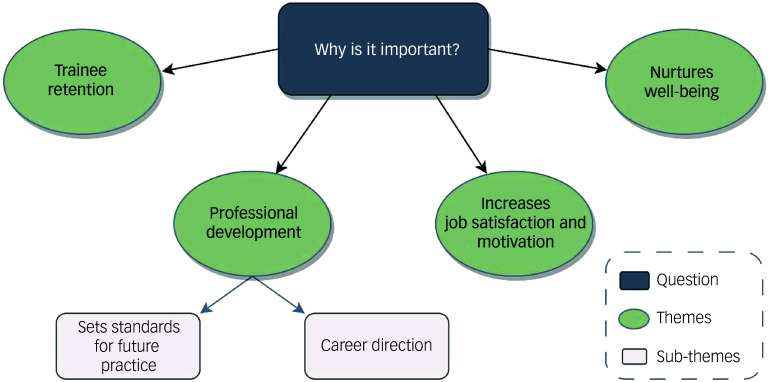
Main themes and sub-themes of ‘Why is it important to have a positive placement experience?’

A positive placement experience is important for trainees because it nurtures their well-being, (increases their job satisfaction and motivation and is conducive towards their professional development, ultimately playing a role in trainee retention (Fig. 2).

### Theme 1: Nurtures well-being

Trainees reflected on the personal impact of their training, feeling that ‘good’ placements allow them to ‘enjoy your job’, with a direct impact on being ‘happy in life more generally’:

‘I think, as well, that being happy in work, being able to and learn how to manage our time, […] has a direct impact on our personal lives, our families and on how much pleasure we get outside of work. So, I think that makes you happier’.

Trainees were universally conscious of potential burnout, and valued good placements with ‘time to think’ and ‘flexibility’ as promoting well-being. ‘Feeling valued’ in a placement is also a positive factor in ‘your capacity to cope with your workload’:

‘I suppose the more you enjoy it, the more you’re likely to learn because you’ll put in more. And the more you put in, the more you’ll get out. If you go to work, hating it, I don’t think you’ll probably learn as much […] And it can affect your relationships, friendship, and just your general mood, I think. And if your moods are affected, then you’ll find it difficult to engage in your job. So, it’s a vicious cycle’.

‘… enjoying your job and feeling valued in your job has a clear knock-on effect to your capacity to cope with your workload, and probably to your sort of sense of avoiding burnout …*’*

### Theme 2: Increases job satisfaction and motivation

Because trainees are concerned about their ability to work to their potential and do a ‘good job’, placements providing appropriate infrastructure, psychological safety and sufficient time to think and act are viewed as facilitating this, enabling trainees to engage and concentrate fully on their patients and the training experience:

‘… if people are enjoying their jobs, they are far more likely to put the effort to do the extra bits, to go the extra mile to work alongside the MDT to get involved in stuff that’s going on …’

Going further, trainees enthusiastically described how positive placements with active learning opportunities contributed to ‘job satisfaction’ and helped to maximise motivation and feelings of competence and confidence, allowing them to ‘deliver better care’ and develop ‘transferable skills’ to carry forward into future placements benefitting other patients and services:

‘Because it gives you job satisfaction, it means that you enjoy your day, you feel like you’re getting better and you’re learning and you’re enjoying your relationships with your work colleagues … you don’t count down the hours of trying to get to the end of the day. So, you’re just satisfied..at least for me, that’s a big part of what just helps me feel happy in life more generally. And then it keeps the motivation and the passion for the job going, especially when you see other people around you who are enjoying their job and those who are satisfied […] it comes just like a cycle of satisfaction is a positive experience’.

### Theme 3: Professional development

A consistent theme of ‘good’ placements is that trainees can observe ‘good supervisors’ and other members of the team who provide ‘positive role modelling’. Good supervisors are ‘inspirational’ in terms of clinical skills, interpersonal relationships within the team and professionalism, ‘providing you with a model for your future work’ and setting ‘a standard you will carry forward’:

*‘*… it’s providing you with a model for your future work … I have taken away from those good rotations … “I’d like to do things like that. I’d like to kind of encourage this kind of culture in a team”. So hopefully you’re thinking “well, actually, how can I make this a reality when I’m a consultant or when I’m an Spr?” … it gives you ideas of how to be a good psychiatrist, how to be a good leader, how to be a good senior . ..so much you can learn’.

### Theme 4: Trainee retention in psychiatry

As trainees journey through their placement and consider future career options, having a ‘good’ experience’ ‘gives you direction’ and helps in considering ‘what do you want to pursue?’. Trainees may choose an area of psychiatry where they have had a good experience, and a good placement can prove to be a pivotal moment for those unsure or undecided about their future career intentions:

‘… having come off of 2 years of difficult core training I felt so burned out .. I felt physically sick .. due to the relentlessness of the job and also due to not enjoying myself because I did not feel intellectually stimulated … I was a machine churning things out … I felt that I could not carry anymore with training and that I was going to leave psychiatry … and then I started a new placement in February, that had the right balance of autonomy and supervision, where I had time to think … and it totally reinstated my interest in psychiatry and mental health … I also started being able to go out and enjoy my weekends and socialise rather than just spending them to sleep and recover from you know, a really busy stressful work week …To the point that now I’m considering applying directly into higher training when 6 months ago, I was I thought I was going to quit training. So, it changes everything you know, and even in my personal life … I have more energy to see friends and do nice things which just feeds back into my overall sense of wellness …’

## Discussion

Our primary aim was to elucidate the multifaceted components of ‘a good psychiatry clinical placement’ by interviewing final year core trainees in psychiatry at SLaM, to address an important gap in the literature and to inform postgraduate medical education practices in psychiatry, both within our local and the wider context. Overall, our findings highlight the importance of (a) psychological safety, (b) active learning opportunities, (c) strong training infrastructure and (d) nurturing the supervisor–supervisee relationship. These factors collectively underpin effective learning, support trainee well-being and promote job satisfaction and retention – critical issues given current workforce challenges, with high burnout and low morale rates reported among psychiatrists^10^ and across specialties.^5–8^

Our results align with the literature and established expectations. Elzain et al^11^ showed that supervisors’ commitment, reasonable workload and working conditions were the most highly rated items when measuring the positives of educational environments. The General Medical Council (GMC) publication ‘Promoting Excellence’ sets standards for medical education and training in the UK, recognises ‘Learning Environment and Culture’ as one of its five themes and emphasises the importance of the learning environment and organisational culture valuing education and training. Our themes of ’the ideal supervisor’ and ‘the learning environment’ reflect these findings and offer qualitative insights into their breakdown (the ‘what’ as defined by the trainees themselves, and also ‘why’ these are important to them). This breakdown is action based and could inform a clinical placement checklist to assure and maintain high training standards. A checklist could include elements such as protected supervision time, regular well-being check-ins, manageable caseloads, opportunities for active learning, time for reflection, access to essential infrastructure etc. Checklist development could be placement specific and designed to accompany the Placement-Specific Personal Development Plan (PSPDP), serving as a flexible guide tailored to individual contexts rather than a rigid standard.

The notion that a range of factors, from individual qualities of the trainee and supervisors to systemic structures, dynamics and resources, contribute to good placements is intuitive and not entirely unexpected, and corroborates widely held views. However, one potentially surprising insight was the consistently high value placed on the ‘educational alliance’^12^ aspect – specifically, how a supervisor’s genuine concern for trainee well-being (in addition to clinical teaching) significantly bolsters the trainee’s self-confidence, resilience and overall satisfaction. Additionally, we found an emphasis on ‘psychological safety’ in the workplace; although not entirely new, the prominence of this theme among psychiatry trainees was particularly notable and underscores the unique emotional demands of psychiatric practice. Key message: supervisors should be supported to develop strong educational alliances by prioritising relational skills, structured feedback and trainee well-being during supervision.

Building on the trainees’ emphasis on supportive and effective clinical placements, a striking insight emerged regarding the attributes of an ‘ideal supervisor’. Core trainees described this as someone combining clinical expertise, mentorship, genuine concern for well-being and sufficient availability to cultivate a nurturing and trusting relationship as an ‘educational alliance’,^12^ where effective learning and teaching go beyond knowledge transmission to foster a positive relationship. In such a relationship the educator’s support, guidance and feedback are tailored to the learner’s needs and fostering growth, development and the attainment of competence, leading to the trainee’s success – similar to the ideal supervisor characteristics reported in the literature.^13,14^ This suggests a potential consensus on the critical role of the educational alliance in medical training, and the worth of a more holistic approach to education that equally values interpersonal relationships and clinical expertise. Key message: supervisor development programmes should include training on how to build and review such aliances.

In reality, systemic pressures such as limited time, administrative burdens and high service demands make it difficult for supervisors to consistently meet aspirational ideals. The GMC National Training Survey showed that, in 2023 and 2024, 11% of psychiatry trainers were at risk of burnout, reflecting the challenging conditions in which supervisors operate.^15^ Exclusively celebrating the ‘ideal’ supervisor risks setting unrealistic expectations and overlooking these constraints. A more constructive approach may be to identify and promote the conditions, skills and supports that enable supervisors to thrive. Encouragingly, our appreciative inquiry uncovered examples of supervisors who excelled despite these pressures. Future research could explore the strategies and systemic factors that enable such success, informing faculty development and interventions to support supervisor well-being. Key message: placement design and core trainee allocation should take into account supervisor workload, consider regular checks of supervisor well-being and have an infrastructure that allows breaks from their role and avoids unrealistic expectations that risk educator burnout.

‘Good placements’ were deemed invaluable for personal and professional development but, crucially, also for enhancing trainee well-being. While unsurprising, this is significant in an era marked by high burnout rates and reduced morale across medical specialties. GMC’s recent report on workplace experiences in the UK^15^ shows that, given workplace pressures, a significant proportion of trainees are changing their working patterns, with one in five reporting reduction in their working hours. In a recent survey, 13% of doctors practising in the UK reported being very likely to move abroad to practise medicine.^16^ These developments have major implications for workforce planning and the future of medicine in the UK, with psychiatry unlikely to buck these trends. We believe that improving the quality of clinical placements by strengthening some of the characteristics we have identified may contribute to reversing the negative experiences of trainees. Key message: placement organisers should routinely evaluate the trainee experience and proactively strengthen features known to support retention such as autonomy, psychological safety and meaningful learning.

While our findings offer valuable insights, limitations must also be acknowledged. Our study focuses on core trainees within SLaM, and their views and experiences might not be fully representative of those in other training programmes that differ in structure and resources. For example, SLaM is host to national specialised services and, through its links with Kings College and the Institute of Psychiatry, Psychology and Neurosciences (IoPPN), offers a broad selection of clinical and academic opportunities for interested trainees. One of our themes, the cherry on top, captured core trainees’ appreciation of these resources and their impact on their overall training experience, while other themes and sub-themes not specifically related to academia and expert services are expected to be more representative of a larger national cohort of core trainees. Future research could conduct similar studies at a wider regional or national level through cooperation among different training trusts, allowing for a mapping of similarities and differences between academic versus non-academic centres. Another potential limitation of our study is not triangulating data with the supervisors’ perspectives. We plan to conduct a separate study on this, by presenting the current findings to clinical and educational supervisors and eliciting their views and perspectives on core trainees’ responses and ‘what a good clinical placement means’. Additionally, the small number of participants available for member checking, due to many having moved on from SLaM, could have impacted the representativeness of our interpretations, although we believe that our use of alternative reflexivity methods effectively mitigates potential biases and note that two participating members validated our findings.

Future research could expand the scope of our study to explore potential variances between academic and non-academic training centres at both the regional and national level, improving the generalisability of our findings. Additional research exploring supervisors’ views to highlight potential contrasting views, challenges and barriers, as well as the best way to tackle them, would help complete the picture and inform robust standards of practice. Further qualitative studies could explore the success stories to learn from ‘good placements’ and ‘ideal supervisors’, what works and what does not, to apply insights on their success to our practice. Lastly, service evaluation and quality improvements in postgraduate medical education practices are needed in psychiatry to understand what further resources are required and how to best implement these to sustain and improve the quality of clinical placements and training opportunities.

To summarise, our study enhances understanding of the essential elements of a good psychiatry clinical placement from the perspective of final-year core trainees in the SLaM programme. We identify key components, including psychological safety, active learning, robust training infrastructure and supportive supervisor–supervisee relationships, as crucial to fostering a productive training environment. These elements enhance the professional growth and well-being of trainees and positively impact job satisfaction and retention in psychiatry. However, our research is limited to a single academic setting, underscoring the need for further studies in diverse training environments to broaden the applicability of our findings. Future research should aim to incorporate supervisors’ perspectives, examine the balance between ideal and practical supervisory roles and explore the interplay of various factors contributing to the quality of clinical placements. This can develop a more holistic view of the training experience in psychiatry, and improve educational practices and standards across psychiatric training programmes.

## Supporting information

Varvari et al. supplementary material Varvari et al. supplementary material

## Data Availability

Interview transcripts are not publicly available due to the potential for participant identification through responses but, upon request, excerpts related to the codes of interest can be provided.

## Supplementary material

The supplementary material is available online at https://doi.org/10.1192/bjo.2025.10777
