# Supplementary material for: Characteristics of a good training placement in psychiatry: qualitative interview study of core trainees
Source: BJPsych Open. 2025 Aug 18;11(5):e184. doi: 10.1192/bjo.2025.10777 (PMC12451721; doi:10.1192/bjo.2025.10777)
Supplement: Varvari et al. supplementary material [file S2056472425107771sup001.docx]

**1:1 Interview Schedule**

What makes a Good Psychiatry Clinical Placement? – Insights from core trainees in psychiatry in SLAM NHS Trust

****Only Part 1 is included in the present paper, Part 2 will be published separately.***

**Part 1: Broad definition and views**

Q1: How would you **define** a good clinical placement?

Q2: Think about your most positive experience in a clinical placement. Can you **describe** that experience and explain **what** made it positive and **why?**

Q3: Are there particular **aspects** of a clinical placement that you believe are **crucial** to having a positive training experience? **What are they? And why?**

Q4: What are some **potential benefits** to having a positive clinical placement experience? How might these benefits translate to your **personal and professional development**?

Q5: What have been some of the most positive things that you have heard from **other trainees** about their clinical placement experiences? What made those experiences so successful?

Q6: Are there any particular positive factors that are **relevant only to psychiatry** that contributed to a positive clinical placement experience that you had.

Q7: Think about a positive clinical placement experience, **how did you contribute to its positivity**?

**Part 2: Targeting specific components per expected placement principles**

**Enhancing real world practice skills**

Q8: Think about a clinical placement where you felt you gained practical skills that were relevant to your future practice. Can you describe what made that placement effective in helping you develop those skills.

**Effective supervision**

Q9: Reflecting on your experiences in different clinical placements, can you think of a placement where you felt you received effective supervision? What made the supervision effective in that placement?

**Promoting and fostering a culture of reflective practice and self-directed learning**

Q10: In your opinion, what are some important factors that contribute to a clinical placement fostering a culture of reflection and self-directed learning?

**Developing Patient centred care**

Q11: Think about a clinical placement where you felt you were able to provide patient-centred care. Can you describe what made that placement conducive to providing patient-centred care?

**Learning about professional conduct**

Q12: In your experience, what are some effective methods for teaching professional conduct during clinical placements? Can you describe a placement where you felt you learned important lessons about professional conduct?
